# First inter-laboratory comparison of *Echinococcus granulosus sensu lato* diagnosis in Latin America

**DOI:** 10.26633/RPSP.2019.89

**Published:** 2019-12-09

**Authors:** María Isabel Jercic, Graciela Santillan, Susana Elola, William Quispe Paredes, Lidia B Conza Blanco, Noelia Morel, Rodrigo Villegas, Baldomero Molina Flores, Cesar M Gavidia, Marta Cabrera, Alexandre Guerra dos Santos, Manuel J Sanchez-Vazquez, Melody J Maxwell, Marco A Vigilato, Edmundo Larrieu, Víctor J Del Rio Vilas

**Affiliations:** 1 Instituto de Salud Pública de Chile Instituto de Salud Pública de Chile Santiago de Chile Chile Instituto de Salud Pública de Chile, Santiago de Chile, Chile.; 2 Instituto Nacional de Enfermedades Infecciosas “Carlos G. Malbran,” Instituto Nacional de Enfermedades Infecciosas “Carlos G. Malbran,” Buenos Aires Argentina Instituto Nacional de Enfermedades Infecciosas “Carlos G. Malbran,” Buenos Aires, Argentina.; 3 Comisión Nacional de Zoonosis Comisión Nacional de Zoonosis Montevideo Uruguay Comisión Nacional de Zoonosis, Montevideo, Uruguay.; 4 Instituto Nacional de Salud Instituto Nacional de Salud Lima Peru Instituto Nacional de Salud, Lima, Peru.; 5 Servicio Nacional de Sanidad Agraria Servicio Nacional de Sanidad Agraria Lima Peru Servicio Nacional de Sanidad Agraria, Lima, Peru.; 6 Centro Panamericano de Fiebre Aftosa Pan American Health Organization (PAHO) / World Health Organization (WHO) Rio de Janeiro Brazil Centro Panamericano de Fiebre Aftosa, Pan American Health Organization (PAHO) / World Health Organization (WHO), Rio de Janeiro, Brazil.; 7 Facultad de Medicina Veterinaria Universidad Nacional Mayor de San Marcos Lima Peru Facultad de Medicina Veterinaria, Universidad Nacional Mayor de San Marcos, Lima, Peru.; 8 The Ohio State University The Ohio State University Columbus, Ohio United States of America The Ohio State University, Columbus, Ohio, United States of America.; 9 PAHO/WHO Country Office PAHO/WHO Country Office San Salvador El Salvador PAHO/WHO Country Office, San Salvador, El Salvador.; 10 Escuela de Veterinaria Universidad Nacional de Rio Negro Choele Choel Argentina Escuela de Veterinaria, Universidad Nacional de Rio Negro, Choele Choel, Argentina; 11 School of Veterinary Medicine University of Surrey Surrey United Kingdom School of Veterinary Medicine, University of Surrey, Surrey, United Kingdom.

**Keywords:** Echinococcosis, dog diseases, laboratory proficiency testing, South America, Equinococosis, enfermedades de los perros, ensayos de aptitud de laboratorios, América del Sur, Equinococose, doenças do cão, ensaio de proficiência laboratorial, América do Sul

## Abstract

**Objective.:**

To compare the performance of polymerase chain reaction (PCR) and enzyme-linked immunosorbent assay (ELISA) tests for diagnosing *Echinococcus granulosus* in dog feces among national reference laboratories in Argentina, Chile, Peru, and Uruguay.

**Methods.:**

National laboratories affiliated with the Ministry of Health/Agriculture of each country exchanged panels of 10 positive/negative samples obtained from their regular national surveillance programs in November 2015 – November 2016. All laboratories applied PCR; two also applied ELISA techniques. Sensitivity and specificity were determined for each laboratory and concordance of results among the laboratories was evaluated by Cohen Kappa coefficient.

**Results.:**

Poor concordance (3 of 10 paired comparisons had values of Kappa > 0.4), low sensitivity and specificity across all laboratories, and poor performance of both techniques in detecting *E. granulosus* in canine feces was demonstrated in this study. An ex-post comparison of the laboratories’ test protocols showed substantial heterogeneity that could partially explain poor concordance of results.

**Conclusion.:**

The results underscore the heterogeneity of canine echinococcosis diagnosis across the region and indicate possible sources of variability. Efforts to standardize canine echinococcosis testing must be included in the plan of action for the Regional Initiative for the Control of Cystic Echinococcosis. Future comparisons with fecal samples of known parasite load are needed.

## RESUMEN

**Objetivo.:**

Comparar el rendimiento de los ensayos de la reacción en cadena de la polimerasa y el enzimoinmunoanálisis de adsorción en fase sólida (o ELISA, por su sigla en inglés) para diagnosticar *Echinococcus granulosus* en heces caninas en los laboratorios de referencia nacionales de Argentina, Chile, Perú y Uruguay.

**Métodos.:**

Los laboratorios nacionales, afiliados a los ministerios de salud y agricultura y ganadería de cada país, intercambiaron paneles de diez muestras positivas y negativas obtenidas de sus respectivos programas nacionales de vigilancia desde el mes de noviembre del año 2015 hasta el mismo mes del año siguiente. Todos los laboratorios emplearon la reacción en cadena de la polimerasa y dos emplearon también técnicas de ensayo inmunoenzimático (ELISA). Se determinó la sensibilidad y la especificidad de cada laboratorio y se evaluó la concordancia entre los resultados de los laboratorios mediante el coeficiente kappa de Cohen.

**Resultados.:**

Este estudio descubrió una escasa concordancia (3 de 10 comparaciones de pares obtuvieron valores de kappa > 0,4), una sensibilidad y especificidad bajas en todos los laboratorios y un rendimiento deficiente de ambas técnicas de diagnóstico de *Echinococcus granulosus* en heces caninas. La comparación *ex post* de los protocolos de ensayo de los laboratorios mostró una heterogeneidad sustancial que podría explicar parcialmente la escasa concordancia de los resultados.

**Conclusiones.:**

Los resultados subrayan la heterogeneidad del diagnóstico de equinococosis canina en toda la región e indican posibles fuentes de esta variabilidad. Deben incluirse medidas para estandarizar la prueba de equinococosis canina en el plan de acción de la Iniciativa Sudamericana para el Control de la Equinococosis Quística. En el futuro serán necesarias comparaciones adicionales con muestras fecales con una carga de parásitos conocida.

## RESUMO

**Objetivo.:**

Comparar o desempenho dos métodos de reação em cadeia da polimerase (PCR) e ensaio imunoenzimático (ELISA) no diagnóstico de infecção pelo *Echinococcus granulosus* em fezes de cães entre laboratórios de referência nacional na Argentina, Chile, Peru e Uruguai.

**Métodos.:**

Laboratórios nacionais conveniados ao Ministério da Saúde/Agricultura de cada país participante intercambiaram grupos de 10 amostras positivas/negativas coletadas rotineiramente pelos programas nacionais de vigilância no período de novembro de 2015 a novembro de 2016. Todos os laboratórios empregaram o método de PCR e dois empregaram também o método de ELISA. A sensibilidade e a especificidade dos métodos foram determinadas em cada laboratório, e a concordância dos resultados entre os laboratórios participantes foi avaliada com o coeficiente kappa de Cohen.

**Resultados.:**

Observou-se fraca concordância (3 de 10 comparações pareadas com kappa >0,4), baixa sensibilidade e especificidade e fraco desempenho de ambos os métodos na identificação do *E. granulosus* em amostras fecais de cães nos laboratórios participantes do estudo. Uma comparação retroativa revelou considerável heterogeneidade dos protocolos de análise laboratorial, o que poderia em parte explicar a fraca concordância entre os resultados.

**Conclusões.:**

Os resultados deste estudo apontam para a falta de uniformidade no diagnóstico de equinococose canina em toda a Região e indicam possíveis causas para variabilidade. A padronização da análise laboratorial da equinococose canina deve constar do plano de ação para a Iniciativa Regional para Controle da Hidatidose. Outras comparações de amostras fecais de parasitas conhecidos devem ser realizadas.

Cystic Echinococcosis (CE), a parasitic zoonosis caused by *Echinococcus granulosus,* is a neglected disease endemic in northern Africa, central Asia, western China, southern and eastern Europe, the Mediterranean area, eastern Russia, and southern South America (1, 2). It is currently considered a multi-species complex called *E. granulosus sensu lato* (3) comprising the species *E. granulosus sensu stricto* (genotypes G1/G2/G3), *E. equinus* (genotype G4), *E. ortleppi* (genotype G5), *E. canadensis* (genotypes G6/G7/G8/G10), and *E. felidis* (“lion” strain).

In South America, CE is endemic in parts of Argentina, Bolivia, Brazil, Chile, Peru, and Uruguay. Except for Bolivia, these countries are members of the Regional Initiative for the Control of Cystic Echinococcosis, hereafter the “Initiative.” In a recent report, the Initiative cited nearly 5 000 new human cases each year and compared dog surveillance figures among the five member countries (4 – 6). Such a comparison is only as good as the concordance of diagnostic techniques among countries.

The adult form of *E. granulosus* lives in the intestine of its definitive host—usually a domestic dog—and releases eggs into the environment through the animal’s feces. The eggs, after accidental ingestion by humans or herbivores (intermediate hosts), may lead to the development of the larval stage (cyst). The eggs have diagnostic value and are important for identifying risk areas contaminated with *E. granulosus*. Such identification is critical for the prevention and control of the disease (2).

The diagnosis of canine echinococcosis may be performed using different immunological methods (7 – 17), e.g., enzyme-linked immunosorbent assay (ELISA) for detection of parasite antigens in feces (copro-ELISA), or molecular techniques such as Polymerase Chain Reaction (copro-PCR). Countries of the Initiative are using various in-house tests, but none are commercially available. For instance, some are using a copro-ELISA test with high sensitivity and specificity that is used for research purposes, standardized by Allan and colleagues (7). Other copro-ELISA polyclonal and monoclonal antibodies have been used to directly detect either somatic or excretory/secretory (ES) antigens. A sensitivity of 78% – 100% and genus specificity of 85% to > 95% have been reported (18); however, cross-reactions occur with infection by *Taenia hydatigena*, the most common taeniid of dogs in areas where *E. granulosus* is endemic. In addition, a low infection burden of less than 50 – 100 worms may produce false negative results to copro-ELISA (18). For copro-PCR, Abbasi and colleagues (9) and Cabrera and colleagues (11) standardized the first test designed for specific detection of *E. granulosus* G1 infection in dogs. The test showed 100% diagnostic sensitivity and specificity, but it was unable to differentiate the genotypes of *E. granulosus*. A copro-PCR specific for *E. granulosus sensu stricto* (G1 genotype) was subsequently developed by Stefanić and colleagues (17).

Both copro-ELISA (8, 15) and copro-PCR (11) tests are currently used for *E. granulosus* surveillance by countries in South America to establish baseline information, monitor program impact, and identify disease hotspots. Due to cost and feasibility concerns, the copro-ELISA test is recommended for screening, and the copro-PCR for confirmatory purposes (18). However, each country has implemented the assays according to availability of laboratory supplies, access to detailed protocols, and local infrastructure. The resulting lack of standardized diagnostic tests throughout this geographic area constitutes an important limitation for the deployment of a regional surveillance system.

In support of efforts by the Initiative and the PAHO Center for Foot-and-Mouth Disease (PANAFTOSA) to enhance and standardize laboratory diagnostic capabilities in the Region of the Americas, this paper presents results of the first inter-laboratory exercise with five national reference laboratories in South America. These five laboratories are affiliated with the Ministry of Health and/or Ministry of Agriculture of their respective countries—Argentina, Chile, Peru, and Uruguay. Although Brazil also has a national reference laboratory for *E. granulosus*, it had not developed a copro-PCR or copro-ELISA at the time of this study.

The objective of this study was to evaluate the PCR and ELISA diagnostic tests performed to detect *E. granulosus* in dog feces and to compare the results of each country’s laboratories. In addition, each of the laboratory’s Standard Operating Procedures (SOPs) for PCR and ELISA were reviewed to identify possible sources of variability. Ultimately, the aim was to provide baseline knowledge for the regional standardization of SOPs for the diagnosis of *E. granulosus* in dog feces.

## MATERIALS AND METHODS

Five national reference laboratories took part in the exercise: 1. National Institute of Infectious Diseases (INEI/ARG), Buenos Aires, Argentina; 2. National Institute of Health (INS/Chile), Santiago de Chile, Chile; 3. National Institute of Health (INS/Peru), Lima, Peru; 4. National Agrarian Quality Service (SENASA/Peru), Lima, Peru; and 5. Zoonoses National Commission (CNZ/URU), Montevideo, Uruguay. All five laboratories used copro-PCR tests; Laboratories 1 and 5 also used copro-ELISA tests.

### Dog fecal samples

Except for Uruguay, fecal samples were collected as part of each country’s regular surveillance activities, and thus originated from natural infections. Uruguay sent samples from experimentally-infected dogs. Four laboratories submitted 10 frozen 1 mL-fecal samples (5 positive and 5 negative) to INS-Chile; a fifth laboratory provided 3 positive and 7 negative samples. Samples from four laboratories were fixed in 70% methanol; the fifth used phosphate buffered saline (PBS) -1% formaldehyde. At INS/Chile, all samples were homogenized, aliquoted, coded, and redistributed to the participating laboratories as a single panel consisting of 40 samples (10 from each one of the other four laboratories).

Laboratories confirmed the status (positive/negative) of their samples as follows: INS/CHI by sequencing; INEI/ARG by microscopy and PCR; CNZ/URU by necropsy; and INS/Peru and SENASA/Peru by PCR. Each laboratory shared the true status of its samples only with PANAFTOSA.

### Laboratory diagnosis of dog fecal samples

Each of the five laboratories analyzed the panel of 40 samples using PCR and/or ELISA according to its SOPs. For copro-ELISA, Argentina used the protocol described by Guarnera and colleagues (8); Uruguay used the one described by Morel and colleagues (16). For copro-PCR, the laboratories used either the technique described by Cabrera and colleagues (12) or that of the European Union Reference Laboratory for Parasites (20), specifically:

#### Copro-ELISA test (Argentina laboratory).

Fecal samples were mixed 1:1 with PBS-TWEEN® 20 (Sigma-Aldrich Inc., Darmstadt, Germany) 0.3% and centrifuged at 3 500 rpm for 30 minutes. The supernatant was collected and frozen at −20°C until processing. Immulon® 2 (ImmunoChemistry Technologies LLC, Bloomington, Minnesota, United States) plates were used for the test; 100 μl of antiechinococcus sp. were placed into each well and the plate incubated at refrigeration (4°C). Each plate was washed three times with PBS pH 7.2/Tween 20/0.1 (P/T) for 5 minutes, and then blocked with PBS pH 7.2 / 0.3% Tween 20 for 1 hour at 37°C. Then, 50 μl of fetal bovine sera and 50 μl of fecal supernatant were added to each well. This was incubated for 1 hr at 37°C in a humid chamber. The plate was washed again, and 100 μl of antiechinococcus sp with peroxidase at 1:5000, diluted in P/T, was added and incubated. Plates were washed and 200 μl of substrate acid (2,2-azino-bis-3-ethylbenzothiazoline-6-sulfonic acid [ABTS]) added. Final incubation was performed for 10 minutes; 200 μl of fluoride acid 0.1 N pH 3.2 was used to stop the reaction. ELISA plates were read at 410 nm using an enzyme immunoassay analyzer iMark™ (Bio-Rad Laboratories Inc., Hercules, California, United States) (8).

#### Copro-ELISA test (Uruguay laboratory).

Stool samples were collected in 1% formaldehyde PBS at a 1:4 volume/volume percentage. Samples were shaken and boiled for 20 min. After centrifugation for 10 min, the supernatants were aliquoted and frozen at -20°C until used. Also, 5 mg/mL of monoclonal IgG was dispensed into ELISA plates and incubated overnight at 4°C. The plates were blocked with 5% non-fat milk (PBS) and washed with PBS-Tween. The plates were further treated with PBS (0.1% bovine serum albumin, 5% sucrose, 0.02% sodium azide), flapped repeatedly against adsorbent paper, and dried in a 40% relative humidity chamber for 4 hr. Samples were analyzed after a 1:2 dilution in PBS; 100 mL/well and were incubated for 1 hr at room temperature. The plates were incubated for 1 hr with a 1:5000 dilution (PBS-T) of a peroxidase conjugated rabbit polyclonal antibody to mouse IgG, washed and developed with 100 mL/well of the substrate solution (0.4 mL of a 6 mg/mL of dimethyl sulfoxide solution), and revealed with tetramethylbenzidine, 0.1 mL (of 1% H2O2 in water in a total of 25 mL of 0.1 mole citrate acetate buffer pH 5.5), and incubated for 15 min at room temperature with shaking. The enzymatic reaction was stopped after 15–20 min by the addition of 50 mL of 2 mole sulfuric acid. The absorbance at 450 – 600 nm was read in a microtiter plate reader (15).

#### Copro-PCR assay (Argentina laboratory).

DNA extraction was performed with 1 volume of chloroform: isoamyl alcohol (1:24), vortexing 10 min and centrifuging at room temperature for 5 min at 12 000 g. Then it was precipitated in 0.6 volume of isopropanol (approximately 450 μl) overnight at 4°C, then concentrated by centrifugation at 12 000 g at room temperature for 5 min, washed with cold ethanol 70% without mixing, and centrifuged for 5 min. The supernatant was removed and the pellet was air dried. The DNA was diluted in 50 μl of Milli-Q® (Merck KGaA, Darmstadt, Germany) water and quantified by 1% agarose gel electrophoresis and ultraviolet fluorescence in the presence of ethidium bromide and quantitation markers. The sample was maintained at 4°C until used (11).

For PCR, alignment of the following mitochondrial cytochrome c oxidase subunit 1 sequences was performed: *E. granulosus* G1, G2, G4, G5, G6, and G7 genotype; *E. vogeli; E. oligarthrus; E. multilocularis; Taenia hydatigena*; and *Taenia crassiceps*. Echinococcus specific nucleotides were identified from the above alignment. The sequence of the forward primer was 5’-TCATATTTGTTTGAGKATYAGTKC-3’; the 3’ cytosine being present in the *E. granulosus* strains, *E. vogeli,* and *E. oligarthrus*. The sequence of the reverse primer was 5’-GTAAATAAMACTATAAAAGAAAYMAC-3’; the 3’cytosine being present in only the four Echinococcus species tested. The primers were developed in INEI/ARG (11).

These primers were expected to obtain an amplification product of 285 bp long, with only genomic DNA from *E. granulosus*, *E. oligarthrus*, and *E. vogeli*. The reaction mixture contained 5 μl (approximately 10 ng) of DNA, 1 U of Taq DNA polymerase, 10 mM Tris-HCl pH 9.6, 50 mM KCl, 1.5 mM magnesium chloride, 0.1% Triton, 25 mM for each deoxynucleotide triphosphate and 0.2 uM for each primer. The reaction conditions were: denaturation for 3 min at 94°C, 39 cycles of a denaturation step at 94°C for 1 min, an annealing step at 50°C for 1 min, an elongation step at 72°C for 1 min, and final elongation at 72°C for 3 min. The amplification products were run in 2% agarose gel electrophoresis using 100 bp ladder as a molecular size marker to determine the fragment length. A MyCycler™ thermal cycler (Bio-Rad Laboratories Inc., Hercules, California, United States) was used (11).

#### Copro-PCR assay (Chile, Peru, and Uruguay laboratories).

This methodology was described by Stefanić (17). Isolation of DNA followed the kit manufacturer’s instructions (QIAamp DNA Stool Kit,™ Qiagen, Hilden, Germany). The primer pair for amplification of DNA of *E. granulosus* G1 was chosen from the sequence of the mitochondrial 12S rRNA gene (GenBank accession no. AF297617; primer sequences Eg1f, 5’-CAT TAA TGT ATT TTG TAA AGTTG-3’; Eg1r, 5’-CACATC ATC TTA CAA TAA CAC C-3’) yielding an amplicon of 255 bp. The primers used were CO1.F TTTTTTGGCCATCCTGAGGTTTAT 24 bp and CO1.R TAACGACATAACATAATGAAAATG 24 bp of Thermo Fisher Scientific™.

Amplification reactions were prepared in total volumes of 100 μl consisting of PCR buffer (50 mM KCl, 20 mM TRIS-HCl pH 8.4, 2.5 mM MgCl2, 0.5% Tween 20), 0.2 mM of each dNTP (using dUTP instead of dTTP), 1 mole of each primer, and 0.5 U uracil DNA glycosylase (UDG; Gibco BRL/Life Technologies, Gaithersburg, Maryland, United States). After 10-min incubation steps at 37°C and 94°C (to inactivate the UDG), 2.5 U Taq polymerase (Platinum™ Taq DNA Polymerase Invitrogen™) were added using a ‘‘hot start.’’ Forty cycles of 30 s at 94°C, 30 s at 53°C, and 45 s at 72°C were performed in a T100™ (Bio-Rad Laboratories Inc., Hercules, California, United States) with a final extension at 72°C for 10 min.

The five laboratories sent their results (40 samples from each laboratory) to PANAFTOSA via an online template. In addition, the laboratories completed an online questionnaire describing current test protocols. The results of the exercise and responses to the questionnaires are presented anonymized.

There were no major incidents reported regarding the logistics of delivering samples to the laboratories, but there were delays, in some cases several months, due to required documentation and border controls. Samples remained frozen during this time and were delivered by courier once the issues were resolved.

### Statistical analyses

The sensitivity (Se) and specificity (Sp) for copro-PCR and copro-ELISA were computed for each participating laboratory, together with the 95% confidence intervals (95% CI), assuming the data were obtained by binomial sampling (20, 21). The true status of each laboratory’s samples was reported per their SOPs, as previously described. The concordance of results among laboratories was evaluated by Cohen Kappa coefficient (21, 22).

### Ethics

This study was not submitted to a research ethics committee because no personal data was handled. Biological samples were all obtained according to each the country’s surveillance standards and regulations.

## RESULTS

### Diagnosis by copro-PCR

**FIGURE 1.**
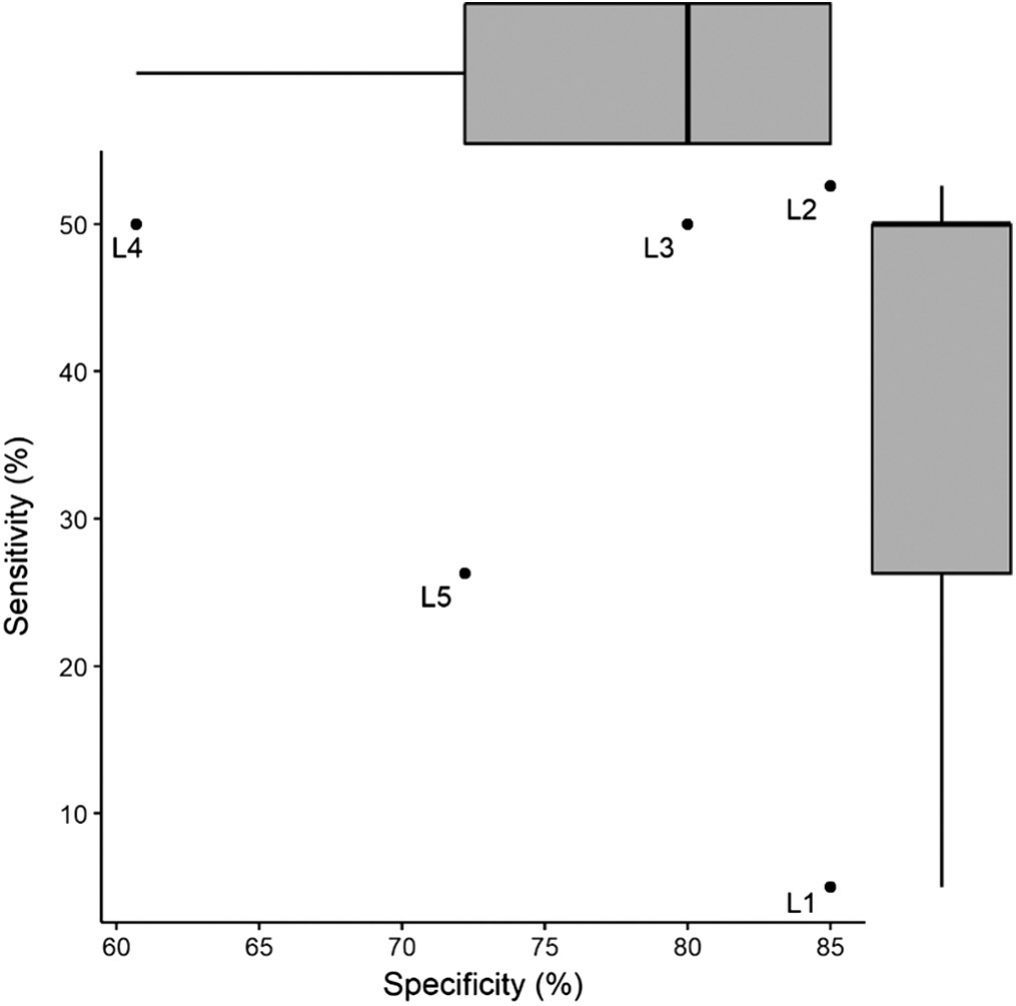
Scatterplot of sensitivity (y-axis) by specificity of diagnosis by copro-PCR for five laboratories in Latin America

**Note:** The sensitivity (y-axis), or the true positive rates and the specificity (x-axis), or the true negative rates, for each laboratory are shown with an open circle. A laboratory correctly identifying all samples would be found in the upper right corner.

***Source:*** Prepared by authors from the study data.

The aggregated Se and Sp for the five laboratories was 35.6% (95%CI = 25.7 – 46.3) and 75.5% (95%CI = 66.2 – 83.3), respectively (Figure 1).

Samples from Laboratory 2 returned the best results (Se 52.6%, 95%CI = 28.9 – 75.6; and Sp 85%, 95%CI = 62.1 – 96.8). Laboratories 3 and 4 returned the best Se values, while Laboratory 5 showed perfect Sp in all samples—although it returned the lowest Se values of the five laboratories (Table 1).

### Diagnosis using copro-ELISA

Laboratory 1 showed low Se and Sp, while Laboratory 5 was unable to detect any positive samples from 3 of the 4 laboratories from which it received samples (Table 2).

Inter-laboratory agreement (Cohen Kappa) for copro-PCR was poor in general. However, Laboratories 3 and 4 showed moderate agreement (Kappa: 0.47; *P* < 0.01), as did Laboratories 4 and 5 (Kappa: 0.50; *P* < 0.001), and Laboratories 2 and 3 (Kappa: 0.58; *P* < 0.001) (Table 3).

## DISCUSSION

The general level of concordance between the participating laboratories was low and showed substantial variability. While some laboratories correctly identified all the samples, the aggregated Se of the exercise was very low at 35.6%. The aggregated Sp (75.5%) was better, but still led to a substantial number of false positives.

The poor results could be due to differences in the laboratories’ test protocols. For copro-PCR, and specifically for the reagents used for DNA extraction, four laboratories used the same kit, but two made modifications to the original manufacturer´s protocol. Using different primers would affect the observed results and use of different DNA stains could modify sensitivity. This is the case for Laboratories 1 and 3, which used ethidium bromide, while the others used another fluorescent nucleic acid dye (13). This variability could have resulted in different detection thresholds that might have excluded animals with low infection loads, resulting in inconsistent identification across the five laboratories.

There were also protocol differences for copro-ELISA. Laboratory 5 was the only one that used monoclonal antibodies, which might explain the disagreement observed, or it could be due to use of formaldehyde for preservation of the samples. It is difficult to conclude what reagent, equipment, or step in the protocol might generate differences among the diagnoses.

A wide array of other factors may have contributed to the observed variability. The original condition of samples followed country-specific standards, and therefore, their quality, volumes, and conservation differed. The age of the samples, cross-reactions with parasitic co-infections, and critically, the incorrect identification of “true” positive and negative samples are possible sources of variability. Note that greater agreement was observed from those samples previously characterized by sequencing. Clearly, future exercises should be performed on a fully sequenced panel.

Despite longstanding CE control programs in some of the participating countries—Argentina, Chile, and Uruguay—external quality assurance mechanisms are not in place (4). These study results reveal possible misclassification of cases, which has potential policy implications, such as diverting resources to treat dogs in areas mistakenly considered endemic.

At the regional level, the variability of the results currently limits valid comparison between countries and undermines surveillance data aggregated to compile regional epidemiologic reports. It also causes important operational problems that make programs and progress difficult to evaluate and areas with active transmission hard to identify

This is the first inter-laboratory exercise for CE in the Region of the Americas, and to the best of our knowledge, in the world. The exercise, supported by the Initiative, follows the example of other neglected diseases, e.g. rabies (23), where inter-laboratory proficiency exercises are conducted regularly and ensure delivery of quality diagnostic services.

The information provided by the five laboratories through the online questionnaire showed differences in the test protocols, which might explain some of the divergent results between laboratories; for example, using different PCR primers or different protocols for processing and blocking of copro-ELISA tests.

**TABLE 1.** Sensitivity (Se) and specificity (Sp) for the copro-PCR, determined by laboratory processing of the samples and sample source, in five laboratories of Latin America

Samples processed by:		Laboratory 1		Laboratory 2		Laboratory 3		Laboratory 4		Laboratory 5	
		Se	Sp	Se	Sp	Se	Sp	Se	Sp	Se	Sp
Laboratory 1	Estimate	—	—	40	60	20	40	33.3	42.9	20	66.7
	95% CI	—	—	5.3 - 85.3	14.7 - 94.7	0.5 - 71.6	5.3 - 85.3	0.8 - 90.6	9.9 - 81.6	0.5 - 71.6	9.4 - 99.2
Laboratory 2	Estimate	0	100	—	—	60	80	33.3	57.1	50	60
	95% CI	0 - 52.2	47.8 - 100	—	—	14.7 - 94.7	28.4 - 99.5	0.8 - 90.6	18.4 - 90.1	6.8 - 93.2	14.7 - 94.7
Laboratory 3	Estimate	20	80	80	80	—	—	33.3	42.9	20	80
	95% CI	0.5 - 71.6	28.4 - 99.5	28.4 - 99.5	28.4 - 99.5	—	—	0.8 - 90.6	9.9 - 81.6	0.5 - 71.6	28.4 - 99.5
Laboratory 4	Estimate	0	60	60	100	100	100	—	—	20	80
	95% CI	0 - 52.2	14.7 - 94.7	14.7 - 94.7	47.8 - 100	47.8 - 100	47.8 - 100	—	—	0.5 - 71.6	28.4 - 99.5
Laboratory 5	Estimate	0	100	25	100	20	100	100	100	—	—
	95% CI	0 - 52.2	47.8 - 100	0.6 - 80.6	47.8 - 100	0.5 - 71.6	47.8 - 100	29.2 - 100	59.0 - 100	—	—

***Note:*** Laboratories are the following: 1. National Institute of Infectious Diseases (INEI/ARG), Argentina; 2. National Institute of Health (INS/Chile), Chile; 3. National Institute of Health (INS/Peru), Peru; 4. National Agrarian Quality Service (SENASA/Peru), Peru; and 5. Zoonoses National Commission (CNZ/URU), Uruguay.

***Source:*** Prepared by authors from the study data.

**TABLE 2.** Sensitivity (Se) and specificity (Sp) for the copro-ELISA, computed by laboratory processing of the samples and the sample source, in five laboratories in Latin America

Samples processed by:		Laboratory 1		Laboratory 2		Laboratory 3		Laboratory 4	
		Se	Sp	Se	Sp	Se	Sp	Se	Sp
Laboratory 1	Estimate	—	—	40	40	20	20	33.3	42.9
	95% CI	—	—	5.3 - 85.3	5.3 - 85.3	0.5 - 71.6	0.5 - 71.6	0.8 - 90.6	9.9 - 81.6
Laboratory 5	Estimate	0	60	60	100	0	80	0	71.4
	95% CI	0 - 52.2	14.7 - 94.7	14.7 - 94.7	47.8 - 100	0 - 52.2	28.4 - 99.5	0 - 70.8	29.0 - 96.3

***Note:*** 1. National Institute of Infectious Diseases (INEI/ARG), Argentina; 2. National Institute of Health (INS/Chile), Chile; 3. National Institute of Health (INS/Peru), Peru; 4. National Agrarian Quality Service (SENASA/Peru), Peru; and 5. Zoonoses National Commission (CNZ/URU), Uruguay.

***Source:*** Prepared by authors from study data.

**TABLE 3.** Kappa coefficient for the copro-PCR results across five laboratories in Latin America

Laboratory	Laboratory 1		Laboratory 2		Laboratory 3		Laboratory 4		Laboratory 5	
	Kappa	*P* value	Kappa	*P* value	Kappa	*P* value	Kappa	*P* value	Kappa	*P* value
Laboratory 1	—	—	-0.25	0.96	-0.04	0.62	-0.16	0.88	-0.2	0.93
Laboratory 2			—	—	0.51	<0.001	0.26	0.03	0.21	0.06
Laboratory 3					—	—	0.38	<0.01	0.03	0.41
Laboratory 4							—	—	0.34	<0.01
Laboratory 5									—	—

***Note:*** 1. National Institute of Infectious Diseases (INEI/ARG), Argentina; 2. National Institute of Health (INS/Chile), Chile; 3. National Institute of Health (INS/Peru), Peru; 4. National Agrarian Quality Service (SENASA/Peru), Peru; and 5. Zoonoses National Commission (CNZ/URU), Uruguay.

***Source:*** Prepared by authors from the study data.

### 

#### Limitations.

The study limitations were important and included variability in laboratory techniques and protocols, differences in sample type and quality, and unconfirmed/unverified sample status.

### Conclusions

This activity was cost-effective and of unquestionable importance. The heterogeneity of canine echinococcosis diagnosis across South America and the sources of important variability merit further attention and research. The Initiative, prompted by these study results, is considering a second comparison exercise, this time with samples of known parasite load, subjected to more standardized sample processing. The Initiative plans to develop surveillance standards that operationalize the Plan of Action for the Elimination of Neglected Infectious Diseases and Post-elimination Actions, including CE (24).

## Author contributions.

VJDRV conceived the original idea. MIJ, GS, SE, WQP, LBCB, NM, and MC collected the data. RV, BMF, CMG, AGDS, MJSV, MJM, MAV, EL, and VJDRV planned the experiments, analyzed the data, and interpreted the results. MIJ, CMG, EL, and VJDRV wrote and reviewed the paper. All authors reviewed and approved the final version.

## Disclaimer.

Authors hold sole responsibility for the views expressed in the manuscript, which may not necessarily reflect the opinion or policy of the *RPSP/PAJPH* and/or PAHO.
